# Local infiltration analgesia is not improved by postoperative intra-articular bolus injections for pain after total hip arthroplasty: A randomized, double-blind, placebo-controlled study with 80 patients

**DOI:** 10.3109/17453674.2015.1081340

**Published:** 2015-11

**Authors:** Karen V Andersen, Lone Nikolajsen, Henrik Daugaard, Niels T Andersen, Viggo Haraldsted, Kjeld Søballe

**Affiliations:** 1 Departments of Orthopedic Surgery; 2 Anesthesiology, Aarhus University Hospital, Aarhus; 3 Biostatistics Section, Department of Public Health, Aarhus University, Aarhus; 4 The Lundbeck Foundation Center for Fast-Track Hip and Knee Arthroplasty, Denmark

## Abstract

**Background and purpose —** The effect of postoperative intra-articular bolus injections after total hip arthroplasty (THA) remains unclear. We tested the hypothesis that intra-articular bolus injections administered every 6 hours after surgery during the first 24 hours would significantly improve analgesia after THA.

**Patients and methods —** 80 patients undergoing THA received high-volume local infiltration analgesia (LIA; 200 mg ropivacaine and 30 mg ketorolac) followed by 4 intra-articular injections with either ropivacaine (100 mg) and ketorolac (15 mg) (the treatment group) or saline (the control group). The intra-articular injections were combined with 4 intravenous injections of either saline (treatment group) or 15 mg ketorolac (control group). All patients received morphine as patient-controlled analgesia (PCA). The primary outcome was consumption of intravenous morphine PCA and secondary outcomes were consumption of oral morphine, pain intensity, side effects, readiness for hospital discharge, length of hospital stay, and postoperative consumption of analgesics at 3, 6, and 12 weeks after surgery.

**Results —** There were no statistically significant differences between the 2 groups regarding postoperative consumption of intravenous morphine PCA. Postoperative pain scores during walking were higher in the treatment group from 24–72 hours after surgery, but other pain scores were similar between groups. Time to readiness for hospital discharge was longer in the treatment group. Other secondary outcomes were similar between groups.

**Interpretation —** Postoperative intra-articular bolus injections of ropivacaine and ketorolac cannot be recommended as analgesic method after THA.

In recent years, there has been increased interest in wound infiltration techniques with local anesthetics for perioperative and postoperative analgesia. A modification of the technique is high-volume local infiltration analgesia (LIA), which was developed by Kerr and Kohan (2008) for analgesia after total hip arthroplasty (THA) and total knee arthroplasty (TKA). LIA involves high-volume intraoperative infiltration and intra-articular re-injections with a mixture of ropivacaine, ketorolac, and epinephrine. Intraoperative LIA with this mixture has been shown to provide effective pain relief after TKA, but there are only limited data available on the analgesic effect after THA. Studies have shown that LIA is more effective in the treatment of postoperative pain after THA than placebo (Andersen et al. 2007b), epidural anesthesia (Andersen et al. 2007a), intravenous infusion of morphine (Parvataneni et al. 2007), and morphine and ketorolac (Bianconi et al. 2003), but these studies had some methodological insufficiencies and used different LIA techniques and mixtures of drugs, which makes interpretation of the results difficult (Kehlet and Andersen 2011).

It is not clear whether intra-articular postoperative top-up bolus injections or continuous infusion of analgesics through an indwelling catheter have any beneficial effect in prolonging analgesia after THA. Quite large variations in postoperative pain intensities have been reported in previous studies. Some authors have reported low pain intensities with an oral multimodal regimen consisting of acetaminophen, nonsteroidal anti-inflammatory drugs (NSAIDs), and gabapentin, but others have reported high intensities of pain despite the use of similar interventions (Liu et al. 2011, Lunn et al. 2011). Also, it is disputed whether NSAIDs have a specific local effect or whether the analgesic effect is achieved through absorption into the systemic circulation. Thus, the optimal form of postoperative analgesia in THA is still being debated.

We therefore carried out a randomized, double-blind, placebo-controlled study to compare the effect of postoperative intra-articular bolus injections with ropivacaine and ketorolac and the effect of intravenous ketorolac administration. The primary outcome measure was the level of intravenous patient-controlled analgesia (PCA) during the first 24 hours after surgery. Secondary outcome measures were consumption of oral morphine as PCA, intensity of pain at rest and during activity, side effects, time of readiness for hospital discharge, length of hospital stay (LOS), and postoperative consumption of analgesics at 3, 6, and 12 weeks after surgery.

## Patients and methods

### Participants

After obtaining written informed consent, 80 patients were enrolled at the Department of Orthopedic Surgery, Aarhus University Hospital, Denmark. Inclusion criteria were planned primary unilateral THA, age over 18 years, planned spinal anesthesia, and tolerance of study drugs (ketorolac, ropivacaine, and morphine). Exclusion criteria were opioid consumption on a daily basis, rheumatoid arthritis, bleeding disorders, obesity (BMI > 35), and inability to communicate in Danish. A secondary exclusion criterion was intraoperative conversion to general anesthesia.

### Randomization and blinding

A pharmacist generated the allocation sequence using computer-generated randomized numbers (block size 8 and allocation ratio 1:1). Patients were randomized to receive 4 postoperative intra-articular bolus injections of ropivacaine and ketorolac (the treatment group) or saline (the control group) combined with intravenous saline (treatment group) or ketorolac (control group). Allocation concealment was ensured using sequentially numbered, opaque sealed envelopes. The allocation list was stored at the local pharmacy until all the patients had been included and until all the 3-month follow-ups had been completed.

### Medication

Blinding of patients, surgeons, healthcare providers, and outcome assessors was done by using tamper-proof study medication delivered in sequentially numbered cassettes and syringes similar in appearance, weight, smell, and viscosity.

After obtaining consent from a patient, we contacted the hospital pharmacy and the study medication was prepared and delivered on the day of surgery. The study medication consisted of 1 bag with 100 mL ropivacaine (2 mg/mL), 1 mL ketorolac (30 mg/mL), and 0.5 mL adrenaline (1 mg/mL) combined with 1 cassette containing either 38 mL ropivacaine (10 mg/mL) and 2 mL ketorolac (30 mg/mL) (treatment group) or 40 mL sodium chloride (9 mg/mL) (control group) and 4 2-mL syringes with either 2 mL sodium chloride (9 mg/mL) (treatment group) or 1.5 mL sodium chloride (9 mg/mL) and ketorolac (30 mg/mL) (control group).

### Anesthesia and surgical technique

As premedication, oral paracetamol (2,000 mg) was given 2 hours before anesthesia. Cefuroxime (1.5 g) was administered before surgery and 6, 12, 18, and 24 hours after surgery. Tranexamic acid (10 mg/kg) was given at the start of surgery and 3 hours afterwards. Spinal anesthesia was induced at the L2–L3 level by using a 25G spinal needle with a dose of 3 mL bupivacaine (5 mg/mL). 3 specialist orthopedic surgeons undertook the procedure according to standard surgical routines, with the patient in a lateral position and using a posterolateral approach (Moore). Drains were not used, and free mobilization was allowed immediately after the operation.

All the patients received intraoperative LIA with 100 mL ropivacaine (2 mg/mL), 1 mL ketorolac (30 mg/mL), and 0.5 mL epinephrine (1 mg/mL) using a standardized technique. After completion of acetabular and femoral surgery, 50 mL of the solution was injected into all the exposed tissues. The rest was injected into the external rotators, the gluteus muscles, and the subcutaneous tissues. Before wound closure, a multi-holed epidural catheter was placed intra-articularly with the catheter tip in the subfascial space and anterosuperior to the joint, and connected to an infusion pump (dose 10 mL, lock-out time 6 hours). The patients were transferred to the post-anesthesia care unit and observed for at least 3 hours postoperatively before being returned to the surgical ward.

### Postoperative treatment

Postoperative pain treatment consisted of 4 intra-articular bolus injections of 9.5 mL ropivacaine (10 mg/mL) with 0.5 mL ketorolac (30 mg/mL) (treatment group) or 10 mL sodium chloride (9 mg/mL) (control group) via a catheter (dose 10 mL, lock-out time 6 hours). The intra-articular injections were combined with 4 intravenous injections of 2 mL sodium chloride (9 mg/mL) (treatment group) or 1.5 mL sodium chloride (9 mg/mL) with 0.5 mL ketorolac (30 mg/mL) (control group) initiated 6 hours after surgery and repeated every 6 hours. All patients received intravenous morphine as PCA (1 mg/mL; dose 2.5 mg, lock-out time 10 min) for the first 24 hours after surgery. The intra-articular bolus injections and intravenous injections after 6 and 24 hours were administered by one of the investigators and the remaining 2 boluses and intravenous injections were given by the nursing staff. All patients were treated with oral acetaminophen (1,000 mg) every 6 hours, initiated 4 hours after surgery and continued during the hospital stay. Nausea was treated with intravenous ondansetron (4 mg) (first choice) or metoclopramide (10 mg). No other analgesics, anti-emetics, or sedative drugs were used during the study period. Oral morphine as PCA (5–10 mg) was allowed as rescue analgesic medication from 24 hours after surgery until discharge from hospital. For thromboprophylaxis, an injection of 5,000 IE dalteparin was administered subcutaneously, starting on the day of surgery (i.e. 8 hours postoperatively) with continuation until discharge. All patients received laxatives.

### Outcome measures

The primary outcome measure was consumption of intravenous PCA morphine from 0 to 24 hours after surgery. Consumption of intravenous PCA morphine was also registered from 0 to 6, 0 to 12, and 0 to 18 hours after surgery. Secondary outcome measures were oral PCA morphine consumption from 24 to 72 hours after surgery, postoperative pain intensity scores at rest and during movement-evoked pain (straight-leg raise until 45 degrees and after walking 5 m) from 6 to 72 hours after surgery, side effects (nausea, vomiting, or itching), time to fulfillment of discharge criteria (home readiness), and LOS.

Before surgery, all the patients were instructed in the use of the visual analog scale (VAS; with 0 representing no pain and 100 representing the worst pain possible) and the intravenous PCA pump. The patients used diaries to record pain intensity and side effects. Pain at rest (measured after a 5-min rest period) and pain during straight-leg raise were both recorded on the day of surgery (immediately before bolus injection) and 30 minutes, 1 hour, 2 hours, 4 hours, and 6 hours after injection. Pain during walking was measured the first time the patients walked on the day of surgery. On postoperative day 1, pain at rest and during straight-leg raise was recorded immediately before bolus injection and 30 min, 1 hour, 2 hours, and 3 hours after injection. Pain during walking was measured once in each time period (8 a.m. to 2 p.m. and 2 p.m. to 8 p.m.) On postoperative days 2 and 3, pain intensity scores at rest and during walking were recorded at 8 a.m. and 8 p.m.

Side effects were recorded for the postoperative periods 0–12 hours on the day of surgery and at 8 a.m. and 8 p.m. on postoperative days 1–3. Episodes occurring during the night were recorded in the morning. Nausea and itching were described on a 4-point verbal scale (none, mild, moderate, and severe), and vomiting as the number of times. In order to ensure compliance, all the patients were visited by one of the investigators 6 and 24 hours after surgery and on the third postoperative day, or at discharge (whichever came first). 1 of the investigators also recorded the consumption of intravenous PCA morphine after 6 and 24 hours.

The patient’s ability to meet the discharge criteria (home readiness) was recorded by the nursing staff every afternoon. The discharge criteria were mild pain (VAS < 30 at rest), pain sufficiently controlled by oral analgesics, no evidence of any surgical complications, and being able to maintain personal hygiene, to eat and drink, to get in and out of bed, to sit and rise from a chair, to walk safely with elbow crutches, and to climb stairs. LOS was recorded as actual time to home discharge once the home discharge criteria were fulfilled (where day 0 was the day of operation).

3, 6, and 12 weeks after surgery, each patient received a questionnaire on pain intensity (VAS) at rest and during activity (walking for 15 min during daily activity) and on consumption of analgesics (opioids and NSAIDS) on a daily basis. 3 months after surgery, all the patients came to a control visit at the hospital.

### Statistics

Sample size was calculated through simulation, reaching a power of 84% with a sample size of 40 per group. This was based on existing data on the percentage distribution of 24-hour morphine consumption in patients who received the control treatment and an expected distribution for the treatment group. The percentage distribution was as follows (control group/treatment group): 0 mg morphine, 10%/30%; ≤ 10 mg, 20%/29%; ≤ 20 mg, 30%/23%; ≤ 30 mg, 20%/10%; and > 30 mg, 20%/8%.

Categorical data were analyzed using the chi-squared test or Fisher’s exact test. Data that did not fulfill the assumptions of normal distribution were analyzed with the Mann-Whitney U-test. Results are presented as frequency, mean (SD), median with interquartile-range (IQR), or median difference with quantile regression bootstrap-based 95% confidence interval (CI), as appropriate. The level of significance was chosen to be 0.05. EpiData software version 3.1 (EpiData Association, Odense, Denmark) was used for double data entry, and statistical analysis was performed with STATA software version 10.0.

### Ethics and registration

The study was approved by the Committee on Health Research Ethics, Central Denmark Region (M-20090218), the Danish Medicines Agency (EudraCT no. 2009-016445-25), and the Danish Data Protection Agency. It was registered at clinicaltrials.gov (identifier NCT01344395) and conducted in accordance with the Helsinki Declaration and the guidelines for Good Clinical Practice (GCP), and was monitored by the GCP unit at Aarhus University Hospital.

## Results

271 patients were assessed for eligibility from May 2010 through July 2011; 17 of these patients were participating in another study, 77 declined participation, and 97 patients were excluded based on the exclusion criteria. 80 patients were enrolled (40 in each group), and data for the primary endpoint were registered for all patients. The response rates for self-reported questionnaires 3, 6, and 12 weeks after surgery ranged from 86% to 94% (Figure). The baseline characteristics were similar in the 2 groups (Table 1).

**Figure:**
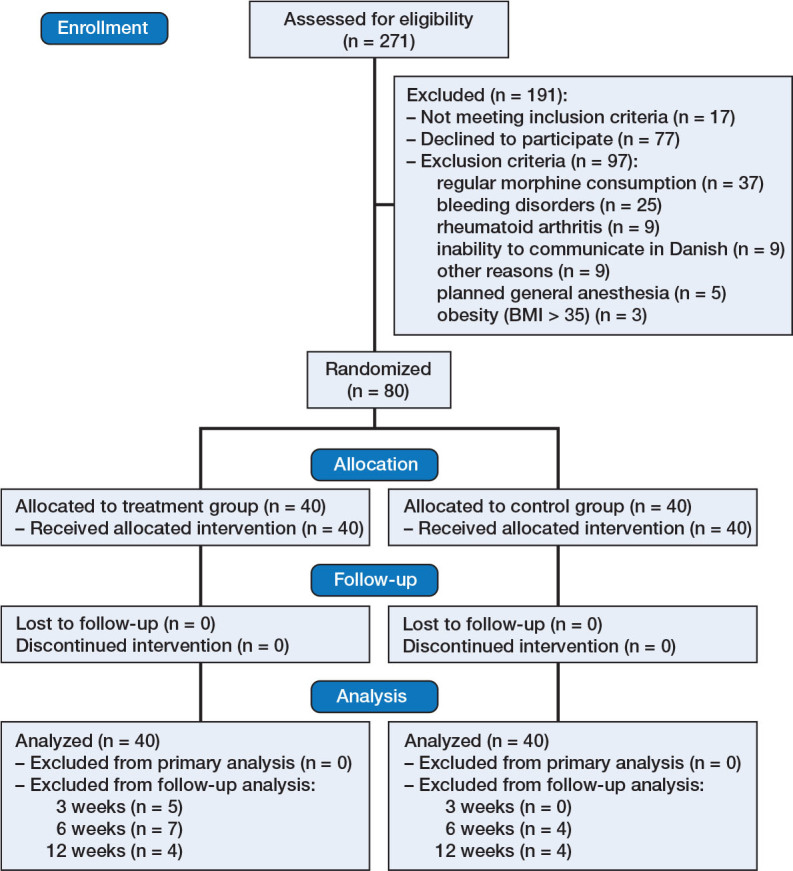
Flow diagram of the study.

**Table 1.** Baseline characteristics. Values are given as number, median (IQR), or mean (SD)

Treatment group (n = 40)	Control group (n = 40)
Gender: M/F	18/22	16/24
Age, years	60 (15)	66 (14)
Height, cm	173 (165–178)	171 (165–175)
Weight, kg	80 (16)	79 (14.8)
BMI	26 (24–30)	27 (25–30)
Hb, mmol/L	8.9 (0.7)	8.8 (0.8)
ASA physical status I/II/III	15/24/1	11/29/0
Duration of surgery, min	70 (17)	71 (19)

There was no statistically significant difference in intravenous morphine consumption between groups (p = 0.9) (Table 2). Movement-evoked VAS pain scores were similar between groups for the first 24 hours; however, beyond 24 hours the late pain scores during walking were statistically significantly higher in the treatment group than in the control group (Table 3). Morphine PCA consumption from 24 hours to 48 hours and from 48 hours to 72 hours after surgery was similar in the treatment group and in the control group: median difference 3.75 mg (95% CI: −19.8 to 19.8; p = 0.4) and 3.75 mg (95% CI: −9.3 to 24.3; p = 0.8), respectively. The median (IQR) length of hospital stay was 2 (2–3) days in both groups. Readiness for hospital discharge was significantly later in the treatment group (2 (2–3) days) than in the control group (2 (1–2) days) (p = 0.03).

**Table 2.** Intravenous morphine PCA consumption in mg. Values are median (IQR) or median difference with 95% CI

Morphine use	Treatment group (n = 40)	Control group (n = 40)	p-value^a^	Median difference (95% CI)
0–6 h	2.5 (0–6.3)	0 (0–3.8)	0.3	2.5 (-0.7 to 5.7)
0–12 h	10 (5–15)	7.5 (5–13)	0.2	2.5 (-1.1 to 6.1)
0–18 h	13 (5–23)	13 (5–18)	0.6	0 (-6.4 to 6.4)
0–24 h	15 (8–29)	14 (5–25)	0.9	1.3 (-10 to 10)

**^a^**Mann-Whitney U-test

**Table 3.** Pain intensity (VAS, 0–100 mm) at rest, when attempting straight-leg raise, and during walking on the day of surgery and on the first postoperative day; also, at rest and during walking on postoperative days 2–3. Values are median (IQR) or median difference with 95% CI

Pain intensity (VAS)	Treatment group (n = 40)	Control group (n = 40)	p-value^a^	Median difference (95% CI)
Day of surgery (Day 0)			
At rest	23 (11–66)	23 (9–53)	0.6	0 (-2.8 to 0.9)
Movement ^b^	39 (18–67)	50 (25–69)	0.6	-11 (-3.7 to 1.7)
Walking	28 (11–55)	20 (11–34)	0.2	8 (-0.3 to 2.9)
Day 1 (8 a.m.)			
At rest	18 (10–43)	17 (6–30)	0.3	1 (-1.1 to 1.5)
Movement ^b^	30 (18–62)	31 (16–62)	0.8	-1 (-2.0 to 1.8)
Walking	15 (9–35)	13 (4–23)	0.2	-2 (-1.0 to 1.2)
Day 1 (8 p.m.)			
At rest	11 (5 –36)	10 (4–31)	0.4	1 (-1.4 to 1.6)
Movement ^b^	24 (12–62)	22 (8–65)	0.4	2 (-1.5 to 1.9)
Walking	21 (9–42)	10 (3–25)	0.01	11 (-0.2 to 2.4)
Day 2			
At rest	15 (6–30)	10 (3–24)	0.1	5 (-0.5 to 1.5)
Walking	28 (16–47)	7 (25–33)	0.08	21 (-1.1 to 1.7)
Day 3			
At rest	17 (6–28)	9 (2–25)	0.09	8 (-0.2 to 1.8)
Walking	23 (13–35)	10 (4–28)	0.01	13 (0.5 to 2.1)

**^a^**Mann-Whitney U-test **^b^**straight-leg raise until 45°

All the side effects recorded (episodes of itching, nausea, and vomiting) were similar between groups, except itching on the first postoperative day, which was significantly better in the control group (p = 0.03) (Table 4, see Supplementary data).

Pain intensity scores and analgesic consumption at 3, 6, and 12 weeks were similar between groups, except pain during walking, which was significantly less in the control group at 6-week follow-up (p = 0.006) (Table 5, see Supplementary data).

There were no major surgical complications. 1 patient in the treatment group was re-admitted to hospital due to dislocation and 1 patient in the control group was re-admitted due to infection (onset < 6 weeks after the original surgery) and successfully treated with a closed relocation and house-cleaning procedure involving debridement of infectious and necrotic tissue, irrigation of the joint, and exchange of the modular components of the hip prosthesis. No signs of deep venous thrombosis or insufficient wound healing were found during the 12-week follow-up period.

## Discussion

### Main findings

The use of postoperative intra-articular bolus injections of ropivacaine and ketorolac did not result in a reduction in morphine consumption after surgery in patients undergoing THA under spinal anesthesia. Also, it was not associated with reduced pain intensity scores at rest or during movement-evoked pain in the early postoperative period (< 24 hours). Indeed, intravenous ketorolac resulted in reduced pain intensity scores during walking in the late postoperative period (1 and 3 days after surgery) and reduced time to home readiness. We chose to use the time for home readiness using objective criteria as an outcome parameter instead of the actual time of discharge, as the latter can be influenced by several non-medical factors. Overall, all comparisons were in favor of the control group and were therefore contrary to our hypothesis.

Our findings are consistent with the results of 2 recent randomized, controlled studies with very different study designs, in which postoperative intra-articular injections or continuous infusions of ropivacaine and ketorolac did not provide superior pain relief after THA. Solovyova et al. (2013) conducted a study in which 105 patients undergoing THA were randomized into 3 groups, each with 35 patients. 2 groups received intraoperative LIA (ropivacaine (100 mg), ketorolac (15 mg), and adrenaline (0.5 mg)) combined with continuous intra-articular infusion (at 5 mL/hour) of either ropivacaine (2 mg/mL) (group I) or saline (group II); the third group was given intraoperative saline infiltration followed by saline infusion at 5 mL/hour for 48 h after the operation. All patients received oral acetaminophen, celecoxib, and pregabalin perioperatively and postoperatively. There were no differences between groups regarding analgesic consumption, pain scores, adverse side effects, or how satisfied the patients were with pain management. Specht et al. (2011) compared postoperative bolus injections to placebo in 60 patients undergoing THA. All patients received intraoperative infiltration with 100 mL ropivacaine (2 mg /mL), 1 mL ketorolac (30 mg/mL) with adrenaline, and 2 bolus injections of the same mixture or saline through a catheter 10 and 22 hours after surgery. All of them received an oral analgesic regimen consisting of acetaminophen and NSAIDs. There was no statistically significant difference between the LIA group and the placebo group postoperatively regarding pain, opioid consumption, or length of hospital stay.

To our knowledge, ours is the first randomized, double-blind study to have investigated the effect of postoperative intra-articular bolus injections and to have kept account of the NSAID component in the mixture in patients undergoing total hip replacement.

There may be some concerns about infiltration and intra-articular use of NSAIDs. In the present study, there were no cases of delayed wound healing, but there was 1 infection in the control group. However, the inclusion of only 80 patients did not provide sufficient statistical power to detect serious but rare adverse events. To our knowledge, no publications on the LIA technique have described an increased incidence of adverse events after local injection of ketorolac.

### Limitations of the study

Some methodological issues must be considered. Firstly, we chose to administer NSAIDs systemically in our control group, but it could be argued that a better effect might have been achieved if ketorolac had been given locally (Convery et al. 1998, Gupta et al. 1999, Romsing et al. 2000, Lavand’homme et al. 2007, Spreng et al. 2010). This administration method could have led to an overestimation of efficacy in favor of the treatment group. However, we found that ketorolac given locally was not more effective, indicating that NSAIDs provide analgesia irrespective of the means of administration.

Secondly, the present study did not have a placebo group that received only saline as LIA for postoperative pain management. We chose an approximate placebo control because we did not find the inclusion of such a group useful, since at least 2 randomized studies have shown that intraoperative LIA with ropivacaine and ketorolac either with or without postoperative top-up bolus injections results in better analgesia than saline (Andersen et al. 2007b, Busch et al. 2010). For example, Andersen et al. (2007b) randomized 40 patients undergoing THA to receive intraoperative infiltration with either saline or 150 mL ropivacaine (2 mg/mL) plus 1 mL ketorolac (30 mg/mL) with epinephrine. The intraoperative infiltration was followed with 1 bolus injection of the study drug through a catheter 24 hours after surgery. Intensity of pain and analgesic consumption were significantly lower in the LIA group.

Thirdly, we chose to administer intraoperative LIA in both groups, although the effect is unclear (Andersen et al. 2011, Liu et al. 2011), and it can be argued that pain treatment with intraoperative LIA could be effective, with an extended postoperative “hangover” pain-reducing effect, making postoperative treatment of little or no importance (Busch et al. 2010). However, 2 recent studies (Andersen et al. 2011, Lunn et al. 2011) found that there was no additional effect of intraoperative LIA with ropivacaine and epinephrine. For example, Lunn and colleagues randomized 120 patients undergoing THA to receive intraoperative infiltration with 150 mL ropivacaine (2 mg/mL) with epinephrine or saline. No additional postoperative bolus injection through a catheter was given. All patients received a multimodal oral analgesic regimen consisting of acetaminophen, celecoxib, and gabapentin. There was no difference in intensity of pain or consumption of rescue analgesic between groups during the whole registration period (2–8 hours after surgery) (Lunn et al. 2011). In their randomized , double-blind, placebo-controlled study, Solovyova et al. (2013) concluded that LIA alone or followed by continuous infusion of ropivacaine as a part of multimodal analgesia after THA provides no additional analgesic benefit or reduction in opioid consumption compared to placebo.

Overall, in studies where a comprehensive multimodal analgesic regimen was not used, LIA appears to have been superior to placebo (Andersen et al. 2007b); however, it does not appear to be of value when used in addition to a perioperative multimodal analgesic regimen of acetaminophen, NSAID (Specht et al. 2011), and gabapentin (Andersen et al. 2011, Lunn et al. 2011, Solovyova et al. 2013). In these 3 trials, pain sores were low, and the extent to which these trials were able to detect a difference (if there was one) is unknown. Unfortunately, the present study also had this lack of assay sensitivity. The power of a trial to detect a large difference in pain intensity (VAS) is high compared with that of a trial in which baseline pain intensity is low, and even a very effective treatment will cause only a small change in pain intensity (Breivik et al. 2000). When baseline pain is mild, a simple, weak analgesic treatment will therefore appear to be as effective as a potent one—with both relieving the mild pain and thus appearing to be equally effective.

In this study, intravenous ketorolac resulted in reduced pain intensity scores during walking in the late postoperative period. However, due to the distribution of data and low pain intensity scores, one must question whether these findings are clinically relevant.

Finally, the external validity of our study can be questioned, as only 80 out of 271 patients were included. Thus, it is difficult to know whether our results are directly relevant to all patients undergoing THA. Unfortunately, this problem applies to many clinical studies. A study investigating the external validity of a randomized trial that included patients undergoing THA showed that non-consenters were older, were less healthy, and were discharged later from hospital (Petersen et al. 2007).

### Conclusion

In summary, our study did not provide statistically significant evidence for superior analgesic efficacy of postoperative top-up bolus injections of ropivacaine and ketorolac compared to a basic analgesic regimen consisting of postoperative oral acetaminophen and intravenous ketorolac. Indeed, intravenous ketorolac resulted in reduced pain intensity scores during walking in the late postoperative period. Because of the low pain intensity scores in general and the large confidence intervals, the clinical relevance of this is uncertain.

Based on the results and the disadvantages of indwelling catheters, we cannot recommend postoperative intra-articular top-up bolus injections as analgesic treatment after THA. Supplementary data

Tables 4 and 5 are available at the Acta Orthopaedica website, www.actaorthop.org, identification number 7718.

## Supplementary Material

Supplementary Material
